# Visualizing chemical states and defects induced magnetism of graphene oxide by spatially-resolved-X-ray microscopy and spectroscopy

**DOI:** 10.1038/srep15439

**Published:** 2015-10-20

**Authors:** Y. F. Wang, Shashi B. Singh, Mukta V. Limaye, Y. C. Shao, S. H. Hsieh, L. Y. Chen, H. C. Hsueh, H. T. Wang, J. W. Chiou, Y. C. Yeh, C. W. Chen, C. H. Chen, Sekhar C. Ray, J. Wang, W. F. Pong, Y. Takagi, T. Ohigashi, T. Yokoyama, N. Kosugi

**Affiliations:** 1 Department of Physics, Tamkang University, Tamsui 251, Taiwan; 2 Institute for Molecular Science, Okazaki 444-8585, Japan; 3 Department of Physics, Indian Institute of Science Education and Research, Bhopal 462066, India; 4 Department of Physics, National Tsinghua University, Hsinchu 300, Taiwan; 5 Department of Applied Physics, National University of Kaohsiung, Kaohsiung 811, Taiwan; 6 Department of Materials Science and Engineering, National Taiwan University, Taipei 106, Taiwan; 7 National Synchrotron Radiation Research Center, Hsinchu 300, Taiwan; 8 Department of Physics, University of South Africa, Johannesburg 1710, South Africa; 9 Canadian Light Source Inc., University of Saskatchewan, Saskatoon S7N 2V3, Canada

## Abstract

This investigation studies the various magnetic behaviors of graphene oxide (GO) and reduced graphene oxides (rGOs) and elucidates the relationship between the chemical states that involve defects therein and their magnetic behaviors in GO sheets. Magnetic hysteresis loop reveals that the GO is ferromagnetic whereas photo-thermal moderately reduced graphene oxide (M-rGO) and heavily reduced graphene oxide (H-rGO) gradually become paramagnetic behavior at room temperature. Scanning transmission X-ray microscopy and corresponding X-ray absorption near-edge structure spectroscopy were utilized to investigate thoroughly the variation of the C 2*p*(π*) states that are bound with oxygen-containing and hydroxyl groups, as well as the C 2*p*(σ*)-derived states in flat and wrinkle regions to clarify the relationship between the spatially-resolved chemical states and the magnetism of GO, M-rGO and H-rGO. The results of X-ray magnetic circular dichroism further support the finding that C 2*p*(σ*)-derived states are the main origin of the magnetism of GO. Based on experimental results and first-principles calculations, the variation in magnetic behavior from GO to M-rGO and to H-rGO is interpreted, and the origin of ferromagnetism is identified as the C 2*p*(σ*)-derived states that involve defects/vacancies rather than the C 2*p*(π*) states that are bound with oxygen-containing and hydroxyl groups on GO sheets.

The mechanism of magnetism in graphene and related materials, even in the absence of *d* and *f* electrons, has gained large interest in recent years^1^,^2^,^3^,^4^,^5^,^6^. Carbon-based materials are considered to be very promising for spintronic applications owing to their weak spin-orbit coupling and their potential to have a long spin life-time^7^. Symmetry breaking at the edges of the sheet, defects/vacancies, the substitution of atoms and hydrogen chemisorption are widely accepted scenarios to elucidate the origin of magnetism in graphene and related materials^3^,^8^,^9^,^10^,^11^,^12^,^13^,^14^,^15^,^16^. Furthermore, in the authors’ earlier report, the *d*^0^ magnetic behavior of ZnO nanocactuses (NCs) and nanowires (NWs) was observed by using X-ray based microscopic and spectroscopic measurements^17^, owing to defects/vacancies in the form of dangling or unpaired O 2*p* states (generated by Zn vacancies) that induced a significant local spin moment between nearest-neighboring O atoms, which finding is also supported by the uneven local spin density that was identified by analyzing the partial density of states (PDOSs) of O 2*p* in ZnO using the local density approximation (LDA)+U method. However, numerous reports have suggested that oxygen-containing [carbonyl (C=O), carboxyl (-COOH), epoxy (C-O-C) etc.] and/or hydroxyl (-OH) groups are the origin of magnetism in graphene and related materials^18^,^19^,^20^,^21^,^22^,^23^,^24^,^25^. Boukhvalov *et al.* suggested that the presence of hydroxyl clusters favors magnetism in graphene and proposed that the most stable magnetic configuration in graphene sheets involves the high-spin hydroxyl groups that are formed on top of wrinkles or ripples^1^. Santos *et al.*^21^ used density functional theory (DFT) to calculate the local spin moments of the carboxyl and hydroxyl groups that are adsorbed on the surface of graphene are 1 and 0.56 μ_B_, respectively. Wang *et al.*^22^ also used DFT calculations to reveal that the hydroxyl group is mostly responsible for ferromagnetism in GO. They further proposed that the presence of two hydroxyl groups bound to non-neighboring carbon atoms that are separated by one carbon atom favors the magnetic moment in GO. However, Bagani *et al.*^26^ presented opposing arguments for various magnetism between GO and rGO, the density of wrinkles in the GO sheet decreased upon chemical reduction at high temperature (600 °C) owing to the removal of many epoxy groups, increasing the number of zigzag edges/edge states, causing rGO to have greater magnetism than GO. The increase in magnetic moment is due to the increase in the number of zigzag edges/edge states after annealing of GO, which are stable with the same spin to minimize the Coulomb repulsion energy. The role of oxygen-containing and hydroxyl groups in inducing magnetization in GO and rGO sheets remains a matter of controversy and, no spatially-resolved experimental measurement to compare chemical states (or oxygen-containing and hydroxyl groups) between wrinkle and flat regions before and after chemical reduction have been conducted. Specifically, no measurement has provided any clear evidence concerning whether the high-spin hydroxyl clusters (or oxygen-containing groups) are truly responsible for the high magnetization on the top of wrinkles on GO sheets, or whether the number of oxygen-containing and hydroxyl groups at the wrinkle and/or flat regions can be reduced by the reduction process, therefore, either to enhance or to reduce the magnetic moment in GO or rGO. Element-specific high-spatial-resolution chemical analysis is a desirable tool for directly examining the role of oxygen-containing and hydroxyl groups in particular regions, to elucidate further the difference between chemical states in specific (wrinkle or flat) regions on the surfaces of GO and rGO sheets. Following our earlier study of the correlation between photoluminescence (PL) properties and electronic structures of GO that have undergone various degree of thermal reduction to form rGOs^27^, in this work, GO, photo-thermally (PT) moderately reduced graphene oxide (M-rGO) and heavily reduced graphene oxide (H-rGO) are thoroughly investigated using X-ray microscopic and spectroscopic techniques to examine the role of oxygen-containing and hydroxyl groups in selected (wrinkle and flat) regions as well as the relationship between the C 2*p* states with defects and magnetic behaviors in GO and rGOs. Based on the experimental results thus obtained in combination with DFT calculations, the various magnetic behaviors as ferromagnetic GO transformed to paramagnetic M-rGO and H-rGO are elucidated.

Since preparing pristine graphene is very difficult, in general, graphene-based nanomaterials are fabricated by firstly synthesizing GO and then reducing it to rGO. To produce samples of M-rGO and H-rGO, the PT-reduction of synthesized GO was carried out under irradiation by a steady-state Xe lamp (500 W) for 3 and 6 hours, respectively. An investigation of magnetic hysteresis (M-H) reveals a gradual variation in ferromagnetic GO to paramagnetic M-rGO, and then to further increase in paramagnetic H-rGO behavior. Synchrotron-based X-ray microscopic and spectroscopic techniques, including Scanning Transmission X-ray Microscopy (STXM), X-ray Absorption Near-Edge Structure (XANES) Spectroscopy, Valence-Band Photoemission Spectroscopy (VB-PES) and X-ray Magnetic Circular Dichroism (XMCD), are used. STXM-XANES is utilized herein because it can identify spatially-resolved (nanometer-scale) electronic structures in selected (wrinkle or flat) regions that are typically extracted using the image masks in STXM^17^,^28^. Element-specific XMCD^29^ provides evidence of ferromagnetic behavior in GO.

## Results and Discussion

**Figure 1:**
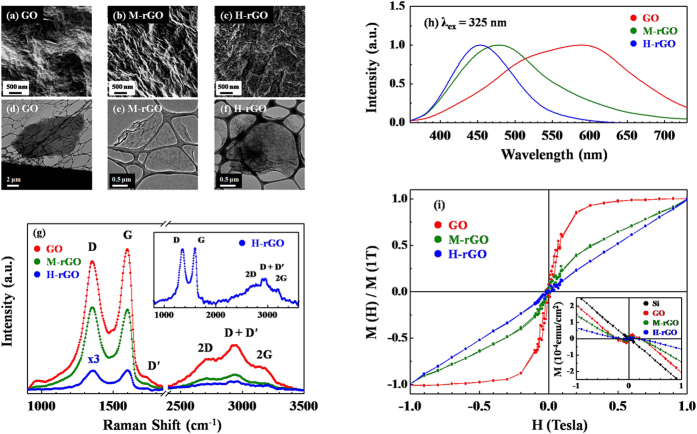
Field Emission Scanning Electron Microscopy images of (**a**) GO, (**b**) M-rGO and (**c**) H-rGO. Transmission Electron Microscopy images of (**d**) GO, (**e**) M-rGO and (**f**) H-rGO. (**g**) Raman spectra of GO, M-rGO and H-rGO; inset magnifies Raman spectrum of H-rGO. (**h**) PL spectra of GO, M-rGO and H-rGO. (**i**) Room-temperature [M(H)/M(1T)]-H curves of GO, M-rGO and H-rGO after subtraction of diamagnetic background that arises from silicon substrate. Inset in Fig. (**i**) plots M-H curves (without background subtraction) of GO, M-rGO and H-rGO.

Figure 1(a–f) display the field emission scanning electron microscopy (SEM) and transmission electron microscopy (TEM) images of GO, M-rGO and H-rGO, respectively. The images reveal a change in surface morphology upon PT-reduction, and that the GO, M-rGO and H-rGO sheets are randomly stacked. Figure 1(g) presents, the Raman spectra of GO, M-rGO and H-rGO samples. The Raman spectrum of GO exhibits four characteristic features, which are the D band at ~1353 cm^−1^, the G band at ~1597 cm^−1^, the 2D band at ~2707 cm^−1^ and the 2G band at ~3184 cm^−1^. A weak feature, D′, is observed at ~1728 cm^−1^, and is attributed to the defects in the samples. PT-reduction significantly reduces the intensities of all features in the Raman spectra of M-rGO and especially H-rGO below those of the GO. The inset in Fig. 1(g) magnifies the Raman spectrum of H-rGO. The feature at ~2920 cm^−1^ arises from a combination of (D + D′) bands and is activated by defects^30^,^31^. Upon PT-reduction, the 2D band of M-rGO and H-rGO becomes lower, and the I_2D_/I_G_ ratio changes. The (I_2D_/I_G_) ratio changes from 0.22 (GO) → 0.15 (M-rGO) → 0.32 (H-rGO). A I_2D_/I_G_ ratio of larger than one typically indicates the formation of bi-layer graphene, whereas an I_2D_/I_G_ ratio of less than one indicates the formation of tri- or multi-layered graphene^32^. For the samples herein, the I_2D_/I_G_ ratios are less than one, suggesting the presence of multi-layered graphene sheets in GO, M-rGO and H-rGO. Meanwhile, the ratio (I_D_/I_G_) of the intensity of the D-band to that of the G-band (which reveals the *sp*^2^*/sp*^3^ ratio) of GO (0.91) is smaller than those of M-rGO (1.02) and H-rGO (0.98). The variation of these I_D_*/*I_G_ ratios is related to structural distortion, surface rippling and wrinkle-structures, which are seen in the SEM images in Fig. 1(a–c), and are formed in the graphene lattice by the restoration of C *sp*^2^ bonds and de-oxidation upon reduction, such that the ratio (I_D_/I_G_) is sensitive to thermal reduction. In contrast to the Raman spectral, the PL spectra of the samples reveal a significant change upon the conversion of GO to M-rGO and H-rGO with various PT-reductions, as presented in Fig. 1(h). Clearly, the PL spectra of GO exhibit asymmetric broad lines from ~400 to 750 nm, whereas those of M-rGO and H-rGO exhibit a single feature that is centered at ~480 nm for M-rGO and ~450 nm for H-rGO. Additionally, the width of the PL lines of M-rGO and, to an even greater extent, H-rGO are smaller significantly than those of GO. This result is consistent with the results obtained in our earlier work in which GO underwent various degrees of thermal reduction to from rGOs^27^. Chien *et al*^33^. demonstrated the change of the original yellow-red PL spectrum of GO to the blue spectrum of rGO and explained that this effect was caused by a reduction in the number of disorder-induced defects in the π-π* gap and the change in *sp*^2^ to *sp*^3^ ratio upon reduction. The original GO in the samples in this study consists of numerous disorder-induced defect states and yield a broad PL spectrum that is centered at long wavelengths (~550–600 nm). Following de-oxidation during the reduction process, the number of defects is reduced, so the M-rGO and H-rGO sheets exhibit blue luminescence. Notably, the X-ray excited orbital photoluminescence and resonant inelastic X-ray scattering, which have been observed in our earlier studies, demonstrated that the PL behaviors are closely related to the density of states (DOS) in the π-π* gap in GO, rGOs and N-doped graphene nanoflakes^27^,^34^. Figure 1(i) plots the normalized magnetization-hysteresis (M-H) curves of GO, M-rGO and H-rGO at room temperature after the diamagnetic contribution from the Si substrate is subtracted; the inset plots the M-H curves of GO, M-rGO and H-rGO and the Si substrate before the diamagnetic Si contribution was subtracted, revealing that the ferromagnetic coercivity and saturated magnetic field of GO were ~150 Oe and 3000 Oe, respectively. The ferromagnetic behavior of GO gradually weakens as the PT-reduction proceeds, exhibiting a paramagnetic behavior for M-rGO and to an even greater extent for H-rGO, although the GO is typically considered to be as being spin-half paramagnetic^35^. The variation of M-H curves in Fig. 1(i), as described in the literature^18^,^19^,^20^,^21^,^22^,^23^,^24^,^25^, if the ferromagnetism of the GO is dominated by the oxygen-containing and/or hydroxyl groups in the GO sheets, especially in the wrinkle regions, then the PT-reduction of GO to M-rGO and H-rGO, must have removed a rising proportion of oxygen-containing and/or hydroxyl groups from GO sheets, so the M-rGO and H-rGO sheets, with fewer oxygen-containing and/or hydroxyl groups, are paramagnetic. If this argument is true, then the magnetism in GO is primarily caused by the C that is π-bonded with oxygen-containing and/or hydroxyl groups, changing the ferromagnetic behavior of GO into the paramagnetic behavior of M-rGO and H-rGO, as the proportion of oxygen-containing and/or hydroxyl groups varies with the degree of PT-reduction. To understand better the origin of ferromagnetic behavior in GO and its gradually giving way to a paramagnetic behavior upon PT-reduction to form M-rGO and H-rGO, STXM-XANES, VB-PES and XMCD are used as follows.

**Figure 2:**
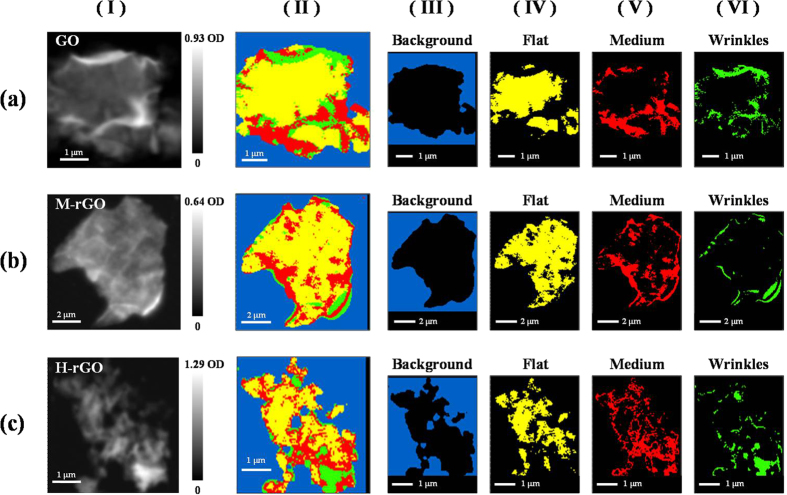
OD images and corresponding stack mapping from STXM images of GO, M-rGO and H-rGO are shown in panels I and II. Panels III-VI present stack mappings from C *K*-edge STXM images of GO, M-rGO and H-rGO, which are decomposed into blue, yellow, red and green regions that are associated with the different thicknesses of samples. Spectra of all samples typically present background (blue), flat (yellow), medium (red) and wrinkle (green) regions.

Figure 2 presents optical density (OD) images (panel I), C *K*-edge STXM stack mappings (panel II) and decomposed STXM mappings (panels III-VI) of the surfaces of randomly selected single sheets of GO, M-rGO and H-rGO. The bright areas in the OD images represent thick regions; dim areas represent thin regions and grey areas represent the regions of intermediate thickness, as observed in GO [panel I(a)], M-rGO [panel I(b)] and H-rGO [panel I(c)], respectively. Based on the OD, the selected regions of GO, M-rGO and H-rGO are typically attributed to wrinkle, medium and flat regions of the GO, M-rGO and H-rGO sheets. As presented in panels I(a)-I(c), the brightest region of H-rGO has a higher average OD (1.29) than does GO (0.93) or M-rGO (0.64), suggesting that the thickest regions were preferably formed in the H-rGO sheets, even though they were the most heavily reduced. The various colors shown in the C *K*-edge STXM stack mapping in panels II(a)-II(c) of Fig. 2 correspond to the randomly varying thickness of GO, M-rGO and H-rGO. The decomposed STXM stack mappings (panels III-VI) are shown in blue (background), yellow (flat), red (medium) and green (wrinkle), which relate directly to the regions of the samples with various thicknesses^17^,^28^. The maps were divided into four regions by principle component analysis (PCA) for cluster analysis, based on spectroscopic differences. The PCA spectrum of each region is the average from all image pixels in that region. The background is shown in blue; the OD or absorbance of the background is nearly zero, corresponding to the near-null intensity of the C *K*-edge STXM spectrum. A more intense average spectrum generally indicates a thicker sample, with the thickness’ increasing form flat, through medium to wrinkle regions. All chemical species in these regions can affect thickness and the thick regions are typically attributed to wrinkle regions of GO sheets. As shown panels IV-VI in Fig. 2, the flat, medium and wrinkle regions are present at random locations on the surface of GO, M-rGO and H-rGO. GO cannot be formed with a perfectly flat geometry because the wrinkle geometry of GO sheets is generally more stable than their flat geometry^36^,^37^, so the formation of wrinkle regions of GO sheets is simply observed in both GO and rGOs. More details concerning STXM-XANES measurement can be found elsewhere^17^,^28^,^38^.

**Figure 3:**
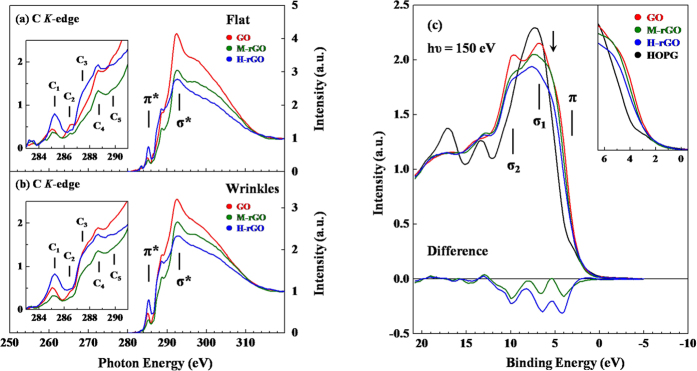
(**a,b**) present C *K*-edge STXM-XANES spectra of GO, M-rGO and H-rGO at flat and wrinkle region respectively. These are the sums of XANES spectra of yellow and green regions of flat and wrinkle regions in panels IV and VI of Fig. 2. Insets magnify 284–290 eV region of STXM-XANES spectra of flat and wrinkle regions. (**c**) Shows the VB-PES spectra of GO, M-rGO and H-rGO with HOPG as reference. Inset magnifies the rising edges of VB-PES spectra. Lower panel displays difference between VB-PES of M-rGO and H-rGO and that of GO.

Figure 3(a,b) present the C *K*-edge STXM-XANES spectra, which are the sum of the XANES spectra from the yellow, flat and green, wrinkle regions in panels IV and VI in Fig. 2, respectively. The difference between the STXM-XANES spectra clarify the relationship between the chemical states that involve oxygen-containing and hydroxyl groups in flat and wrinkle regions and the magnetic behaviors of GO, M-rGO and H-rGO. Based on the dipole-transition selection rules, the C *K*-edge STXM-XANES can be attributed to electron transitions from the C 1*s* core-level to the 2*p* final unoccupied states of GO, M-rGO and H-rGO. The magnified π^*^ region in the insets in Fig. 3(a,b) clearly shows the excited state of C=C (feature **C**_**1**_) at ~285 eV and the wide features of **C**_**2**_-**C**_**5**_ in the range ~286–290 eV between the features π^*^ and σ^*^ of C-C, corresponding to specific oxygen-containing and hydroxyl groups in both flat and wrinkle regions of GO, M-rGO and H-rGO. Although the assignment of these features **C**_**2**_-**C**_**5**_to specific chemical states has been controversial^39^,^40^,^41^,^42^, these features are typically attributed to the chemical states of the C 2*p* that is bound to oxygen and hydrogen atoms, respectively, specific to π^*^(C-OH) at ~286.4 eV, to (C-O-C) at ~287.2 eV, to C=O in the -COOH bond at ~288.5 eV and to C=O at ~290.0 eV^28^,^34^,^43^,^44^. Notably, without considering the polarization effect which can be sensitive to the flat and wrinkle regions measured by STXM, as shown in insets in Fig. 3(a,b), the intensity of features **C**_**2**_-**C**_**5**_in the wrinkle regions [Fig. 3(b)] are nearly constant than those in flat regions [Fig. 3(a)] for GO, M-rGO and H-rGO samples, suggesting the incorporation of oxygen-containing and hydroxyl groups at random in both the wrinkle and the flat regions in GO, M-rGO and H-rGO. However, the feature at ~292 eV is identified as a σ*-resonance feature in both Fig. 3(a,b); its intensity in the wrinkle regions is less than that in the flat regions for GO (maximum feature heights of flat and wrinkle regions are ~4.2 and 3.3, respectively), M-rGO (maximum feature heights of flat and wrinkle regions are ~3.1 and 2.8, respectively) and H-rGO (maximum feature heights of flat and wrinkle regions are ~2.7 and 2.3, respectively). Zhou *et al.* proposed that as the thickness of GO increases, charge redistribution induces more scattering pathways between layers, enhancing the σ*-states transition at the C *K*-edge^28^. Since the wrinkle regions are thicker than the flat regions, the overall intensity of the features associated with the σ*-states is lower in the wrinkle regions than in the flat regions of GO, M-rGO and H-rGO. However, the intensities of the features associated with the σ*-states in both flat and wrinkle regions generally decreased as PT-reduction proceeded from GO to M-rGO and on to H-rGO. In contrast, the intensity of feature **C**_**1**_ of H-rGO exceeds those of GO and M-rGO for both flat and wrinkle regions, indicating the recovery of *sp*^2^ (C-C) bonds upon heavily PT-reduction^27^,^43^. Additionally, the intensities of the features **C**_**2**_**-C**_**4**_ of GO and H-rGO, which are higher than those of M-rGO in flat and wrinkle regions. Surprisingly, the intensities of features **C**_**2**_-**C**_**4**_in the flat (wrinkle) regions of H-rGO are higher than (close to) those of GO. These results reveal that the PT-reduction of GO to M-rGO removed a proportion of oxygen-containing and hydroxyl groups from both flat and wrinkle regions of the GO surfaces. As further PT-reduction yielded H-rGO, the intensities of the features associated with the oxygen-containing and hydroxyl groups in the wrinkle and flat regions, and specifically of features **C**_**2**_ (C-OH) and **C**_**3**_(C-O-C), which are presumed to have a major role in inducing the magnetic moment in GO or rGO as mentioned elsewhere^21^,^22^,^23^,^24^,^25^,^26^, were not significantly reduced. Even H-rGO exhibits more intense features **C**_**3**_ (C-O-C) and **C**_**4**_ (C=O) than does GO in the flat regions. These findings further indicate that as GO is reduced to M-rGO, the oxygen-containing and hydroxyl groups are easily consumed owing to the low activation energy. However, H-rGO exhibits a more intense feature **C**_**1**_ (C=C) in both flat and wrinkle regions than does GO, more intense features **C**_**3**_ and **C**_**4**_ in its flat regions, and almost equally intense features **C**_**2**_-**C**_**4**_ in its wrinkle regions. The intensities of features **C**_**2**_-**C**_**5**_in the wrinkle regions are approximately equal to those in the flat regions of GO, M-rGO and H-rGO. As proposed in the literature^1^, the more oxygen-containing and hydroxyl groups typically decorated the wrinkle regions than the flat regions, inducing high magnetization in the wrinkle regions of GO sheets. Clearly, our spatially resolved STXM-XANES measurements herein do not support the above arguments. The measurements in Fig. 3(a,b) also suggest that the recovery of feature **C**_**1**_ (*sp*^2^ bonds) and the possibly dissolution of some hydroxyl groups to form epoxy and carboxyl groups during the heavily PT-reduction to form H-rGO. The consumption of hydroxyl groups upon heavily PT-reduction to form H-rGO can cause in the activation energy of hydroxyl groups (0.35 eV) to be lower than that of C-H bond (0.55 eV) and the epoxy (C-O-C) group (0.81 eV)^45^. The two step processes that explains why the intensities of features **C**_**3**_ (C-O-C) and **C**_**4**_ (C=O) in the flat regions of H-rGO are higher than those of GO while corresponding intensities of those features in the wrinkle regions are almost same, is proposed, as follows. 1) Two neighboring hydroxyl groups combine and form a water molecule and an oxygen atom and, 2) Further these oxygen atoms react easily with C-H bonds and leave C-O-C and C=O groups on the surfaces of H-rGO. The lower activation energy of the hydroxyl groups relative to that of C-H bonds and epoxy group may be the major reason for the high oxygen coverage of its surface, leading to the formation of the epoxy **C**_**3**_ (C-O-C) and carboxyl **C**_**4**_ (C=O) groups on H-rGO surfaces following heavily PT-reduction^46^. These proposed steps explained why the oxygen-containing and hydroxyl group contents did not decrease monotonically from GO to M-rGO and then to H-rGO. Similar observation have been made in previous studies of the thermal reduction of GO, which revealed the removal of some oxygen-containing groups and the evolution of carbonaceous species in rGOs^34^,^47^,^48^.

As stated above, if the magnetism in GO is mainly determined by the presence of oxygen-containing and/or hydroxyl groups, then the intensity of the corresponding features in the C *K*-edge STXM-XANES spectra should be significantly affected by the transformation from ferromagnetic GO to paramagnetic M-rGO and H-rGO, as seen in the M-H curves in Fig. 1(i). However, according to the insets in Fig. 3(a,b), after heavy PT-reduction, the intensities of features associated with oxygen-containing and hydroxyl groups in the wrinkle regions of H-rGO surfaces are close to those of GO, whereas those in flat regions are higher than those of GO, suggesting that the presence of the C 2*p*(π*) states that are bound with oxygen-containing and hydroxyl groups may not be the main cause of the ferromagnetic behavior in GO. However, the intensities of features associated with the C 2*p*(σ*)-derived states and the above features decrease with the PT-reduction of GO to M-rGO and then to H-rGO, implying the correlation between the numbers of C 2*p*(σ*)-derived states and the transformation of ferromagnetic GO into paramagnetic M-rGO and then H-rGO. This finding combined with the result that the width of the PL line-shapes is significantly reduced from GO to M-rGO and then to H-rGO [Fig. 1(h)], suggests that the reduction in the number of defects as PT-reduction proceeded may importantly affect the magnetic behavior in GO, M-rGO and H-rGO.

Defects that are typically formed by vacancies or dangling bonds close to the ends of graphene and carbon nanotubes are well known to be responsible for the increase in the local DOS in the surface/edge regions^49^,^50^,^51^,^52^,^53^,^54^,^55^, and thereby increase the intensity of those states close to *E*_CBM_ (conduction-band minimum) and *E*_VBM_ (valence-band maximum) or *E*_F_ (Fermi level)^56^,^57^. As observed in Fig. 3(a,b), the density of unoccupied C 2*p*(σ*)-derived states and the above features in GO are clearly greater than those of M-rGO and H-rGO in both flat and wrinkle regions at/above *E*_CBM_ or *E*_F_. A similar enhancement of the density of occupied states at/below *E*_VBM_ or *E*_F_ of the GO relative to those of the M-rGO and H-rGO is also obtained. Figure 3(c) presents the VB-PES of GO, M-rGO, H-rGO and highly ordered pyrolytic graphite (HOPG) for reference. For HOPG, the valence-band spectral feature between ~2 and 12 eV is attributed to C 2*p* states whereas the region between ~12 and 22  eV and higher region are related to C 2*s* and O 2*s* states, respectively. More specifically, the features at ~3 eV and 7 eV are assigned to C 2*p*(π) (solid line) and 2*p*(σ) states^58^,^59^, respectively. Apparently, the GO, M-rGO and H-rGO yield features similar as HOPG, except those related to oxygen-containing groups and some modifications of C 2*p* states. The rising edge of the valence-band shifts to a lower binding energy from GO to M-rGO, to H-rGO and to HOPG, as magnified in the inset of Fig. 3(c). The 2*p*(σ) states in GO, M-rGO and H-rGO splits into two features (indicated by two solid lines): feature (σ_1_) at ~6.8 eV is related to C 2*p*(σ) whereas feature (σ_2_) at ~9.8 eV is attributed to the hybridized state of C 2*p*(σ) and O 2*p*^57^,^58^. The shoulder at ~5.2 eV that is indicted by the arrow is attributed to C 2*p*(*π*-*σ*). The lower panel in Fig. 3(c) displays the difference between the VB-PES of M-rGO and H-rGO and that of GO, and shows that the intensities of spectral features at/below *E*_VBM_ or *E*_F_ of M-rGO and H-rGO are smaller than those of GO. The gradually reduction of the intensity of features C 2*p*(π) and 2*p*(σ) was also observed from GO to M-rGO to H-rGO, suggesting that the numbers of C 2*p*(π) and 2*p*(σ) states decreased by a reduction of the number of defects and/or de-oxidation upon various reduction processes. The intensities of feature C 2*p*(σ*) in the STXM-XANES spectra and feature C 2*p* states in the VB-PES spectra decrease gradually from GO to M-rGO to H-rGO, and the width of the PL line-shapes similarly decrease, supporting the presence of defects on the surfaces of the samples, which may have an important role in changing the magnetic behavior of GO to that of M-rGO and that of H-rGO.

**Figure 4:**
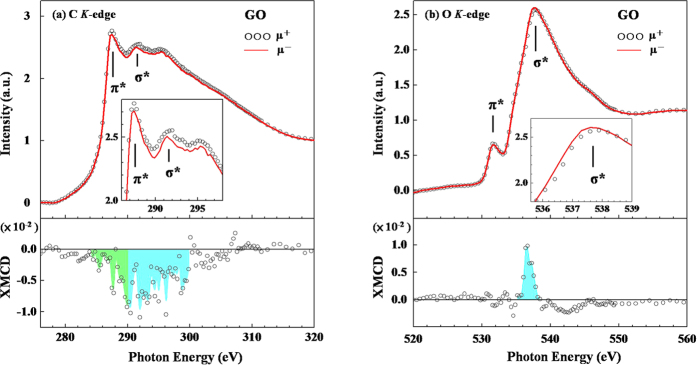
(**a,b**) C and O *K*-edge shows XANES spectra of GO with photo-helicity of incident X-rays parallel (μ^+^) and anti-parallel (μ^−^) to direction of magnetization, respectively. Inset magnifies *π*-*σ* (*σ*) region of C (O) *K*-edge XANES spectra with incident X-rays μ^+^ and μ^−^ to direction of magnetization. Bottom panels present C and O *K*-edge XMCD spectra of GO.

To verify that the C 2*p*(σ*)-derived states involve defects, which play an important role in the magnetism of GO, Fig. 4(a) displays the C *K*-edge XANES spectra of GO, with the photo-helicity of incident X-ray parallel (μ^+^) and anti-parallel (μ^−^) to the direction of magnetization of GO, in an applied magnetic field of ± 1 T (in the opposite direction). As stated above, the C *K*-edge XANES features in Fig. 4(a) within the regions 284–290 eV and 290–300 eV are known to be associated with the C 1*s* → 2*p*(π*) and 1*s* → 2*p*(σ*) transitions, respectively. Notably, the general line-shapes in the C *K*-edge XANES spectra of GO, presented in Fig. 4(a), differ from those of the features in the C *K*-edge STXM-XANES spectra of GO in Fig. 3(a,b). Apparently, the intensity of feature π* exceeds that of feature σ* in Fig. 4(a), whereas Fig. 3(a,b) present a weak feature π* and a broad and strong feature σ*. This difference arises from the fact that the C *K*-edge XANES spectra of GO are highly sensitive to the angle of incident light, as are those of HOPG spectrum^60^,^61^. In this work, the angle of incidence θ of the X-ray between the surface normal and incident light is approximately 70^0^ and 0^0^ for C *K*-edge XANES [Fig. 4(a)] and STXM-XANES [Fig. 3(a,b)], respectively. Notably, the faint but wide-ranging features at ~280–284 eV in Fig. 4(a), may have arisen from the contamination by carbon of the grating optics at the beamline^62^, but, it is generally regarded as contributing equal to the photo-helicity of μ^+^ and μ^−^, so the effects of contamination by carbon can be considered to cancel each other out and not to affect the results of the XMCD analysis. The inset in Fig. 4(a) magnifies the C *K*-edge XANES spectra (in the region 286–298 eV), with the photo-helicity μ^+^ and μ^−^ to the direction of magnetization of GO. The lower panel in Fig. 4(a) displays the C *K-*edge XMCD spectra, (μ^−^ − μ^+^)_/_(μ^+^ + μ^−^). A weak but confirmed magnetic moment is associated with the C 2*p* states in GO at room temperature. Importantly, the intensity of the XMCD features is in the range 284–300 eV at the C *K*-edge of GO, attributed to both C 2*p*(π*)- (green, within the region 284–290 eV) and 2*p*(σ*)-derived states (blue, within the region 290–300 eV). Clearly, the 2*p*(σ*)-derived states are the main contributors to the intensity of XMCD, and the ratio of the integrated blue and green features, presented in the lower panel of Fig. 4(a), is approximately 2.9, indicating C 2*p*(σ*)-derived states are mostly responsible for the magnetism of GO. Still, as presented in the lower panel of Fig. 4(a), the ferromagnetic behavior of GO also involves the C 2*p*(π*)-derived states (green region) bound to oxygen-containing and hydroxyl groups that are observed from the C *K*-edge XMCD results.

As addressed above, magnetic moments of ZnO NCs and NWs have been identified at the O sites, based on O *K*-edge XMCD measurements^17^, owing to the defects in the form of dangling or unpaired O 2*p* states. Besides the fact that the C 2*p*(σ*)-derived states that are associated with defects can be a major factor in determining the net spin polarization in GO. The magnetism of GO may also be caused by the O 2*p*-derived states that are associated with defects at O sites. Figure 4(b) displays the O *K*-edge XANES spectra, with the photo-helicity μ^+^ and μ^−^ to the direction of magnetization of GO (in an applied magnetic field of ±1 T). The lower panel in Fig. 4(b), corresponding to the XMCD feature, lies to the positive side within the range 535–539 eV, which is associated with O 2*p*(σ*)-derived states. Although the chemisorbed oxygen-containing and hydroxyl groups may be responsible for a net spin polarization are commonly present on GO surfaces^43^, the O *K*-edge XMCD spectra of GO reveals that the positive feature is primarily the result of defects associated with in dangling or unpaired O 2*p* states, which induce a local spin moment between nearest-neighboring O atoms, as observed for ZnO NCs and NWs previously^17^. In this study, the C and O *K*-edge XMCD spectra of GO strongly support the claim that the intrinsic *d*^*0*^ magnetism arises from defects. The signs of the C and O *K*-edge XMCD features oppose each other, possibly because the projected orbital contributions of the dangling or unpaired 2*p* states, cause the magnetic moment of the C atoms to align antiparallel to that of the O atoms in the GO layer. However, the integrated intensity of the C *K*-edge XMCD of C 2*p* greatly exceeds that of the O *K*-edge XMCD of O 2*p*, suggesting that the C 2*p*(σ*)-derived states that involve defects dominate the net spin polarization in GO sheets. Furthermore, the negative sign of the C *K*-edge XMCD feature, implying positive orbital magnetic moment on C atoms, also demonstrates the breakdown of the Hund’s 3^rd^ rule since the spin magnetic moment on C atoms is parallel to the external magnetic field and is thus positive, although the less-than-half electron occupation case as C 2*p* may exhibit antiparallel spin and orbital magnetic moments according to the Hund’s 3^rd^ rule. However, such breakdowns were observed in many amorphous/nano systems with induced magnetic moments^63^,^64^,^65^ and few bulk systems with intrinsic moments^66^. The nano-crystalline nature and/or presence/modification of chemical environment of GO possibly the reason for such violation of the Hund’s 3^rd^ rule. It is not surprised that the Hund’s 3^rd^ rule did not fulfilled in our case, vacancy induced magnetism in graphene, based on the following reasons: 1) Local magnetic moment is mainly attributed to dangling *sp*^2^ orbitals and π electrons of the carbon triangle around the defect center. The significant inter-atomic spin-orbit coupling, rather than intra-atomic in the original Hund’s 3^rd^ rule, could be expected in these delocalized orbitals and change the alignment between spin and orbital moments in carbon atoms with induced magnetic moments. However, for O atoms, the small magnetic moment originates from fairly localized electrons on the atomic sites of O which is much similar to the intrinsic magnetic moment in transitional elements (such as 3*d* orbital’s in Mn)^67^. Thus, the Hund’s 3^rd^ rule may apply in this situation. This scenario agrees well with our XMCD finding. 2) In present case, the magnetic properties are dominated by Jahn-Teller (J-T) and exchange effects; therefore spin-orbit effect is essentially weak due to their higher order perturbation^68^,^69^.

**Figure 5:**
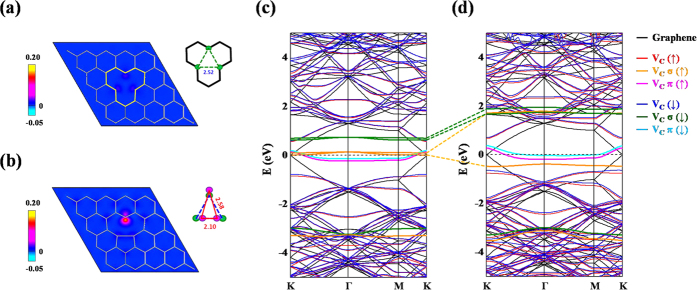
The local (**a**) symmetric structure and (**b**) J-T defect structure around a single vacancy in graphene with corresponding spin-density projections are shown. Local distortions and corresponding C-C distances (in Å) of carbon triangles that surround vacancy centers are highlighted. Spin-polarized electronic band structures of (**c**) symmetric and (**d**) J-T defect structures are also illustrated. Majority and minority spins are indicated by blue and red curves, respectively. Band structures of pristine graphene are denoted as black curves for reference. Fermi level (*E*_F_), indicated as a dashed line, is set to 0 eV for alignment.

Detailed theoretical investigation is conducted to determine the origin of the experimental observations herein. The results of experiments clearly reveal a strong connection between the DOS of the C 2*p*(σ*)-derived states that are related to the defects and the magnetic behavior of GO, rather than C π magnetic moments related to oxygen-containing and hydroxyl groups. Therefore, a simple proposed origin of magnetism of graphene sheets that involves the direct absorption of oxygen-containing and hydroxyl groups was excluded based on both experimental observations herein and recent theoretical studies^70^,^71^. However, as another scenario of the mechanism of magnetism in GO, defects/vacancies are generated more easily by removing C atoms from graphene sheet and generating local magnetic moments that are associated with an imbalance of the graphene sublattice^11^,^72^,^73^. Therefore, the electronic structures of relaxed vacancy-induced symmetric and asymmetric local structures based on DFT calculations are compared here to elucidate the mechanism of defect-induced magnetism in GO. Apparently, the introduction of a vacancy yields a dangling bond on each C atom next to the vacancy. As presented in Fig. 5(a), the symmetric defect configuration, preserving the original equilateral triangular *D*_*3h*_ symmetry with an equal distance (2.52 Å) between each pair of unbonded C atom, which is a metastable structure with a higher formation energy (0.28 eV/vacancy) than that of the optimized asymmetric structure in Fig. 5(b). Similar to a previously obtained theoretical findings^11^,^74^, the calculated optimized structure in Fig. 5(b) herein shows that the vacancy induces a breakdown of the local three-fold *D*_3h_ symmetry of planar graphene to yield *C*_2v_ symmetry around the defect center. Owing to the reconstruction of dangling bonds, the unbonded C triangle is distorted into an isosceles triangle in which one short C-C bond of 2.10 Å is formed and the other two C-C distances are increased to 2.58 Å, as depicted schematically in Fig. 5(b). This J-T distortion forms a unique five-membered ring next to the vacancy, and the dangling bond of the third out-of-plane neighboring C atom is unsaturated. Figure 5(a,b) also plot the calculated contours of spin density projections, defined as the difference between majority and minority spin densities in a real-space representation, for both defect structures. Interestingly, a significant spin density is obtained at the unbonded C atom in the J-T structure, which, along with the non-negligible contribution of two reconstructed C atoms, gives rise to a huge magnetic moment of 1.7 μ_B_. However, the symmetric defect structure with three unbonded C atoms forms only a low-spin configuration with a magnetic moment of 0.37 μ_B_. To understand better this unusual phenomenon, the electronic structures and corresponding PDOS of symmetric and J-T defects are calculated. As shown in Fig. 5(c), the symmetric defect structure destroys the Dirac cone of pristine graphene and forms quasilocalized π states (labeled as *V*_C_ π) in the midgap region indicated by pink (spin up, 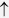) and light blue (spin down, 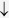) curves. These π electrons are responsible for the magnetism of the symmetric defect configuration. Meanwhile, less dispersive vacancy bands (orange and green curves for 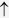 and 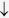, respectively) with σ character (*V*_C_ σ) are located above the *E*_F_ with a exchange splitting of ~0.6 eV. However, in the band structure of J-T defect structure presented in Fig. 5(d), the orbital reconstruction induces a large splitting of σ states (approximately 2 eV) and shifts a separated σ majority state (*V*_C_ σ 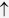) below *E*_F_. This J-T splitting contributes an extra 1 μ_B_ to the local magnetic moment in the asymmetric case.

**Figure 6:**
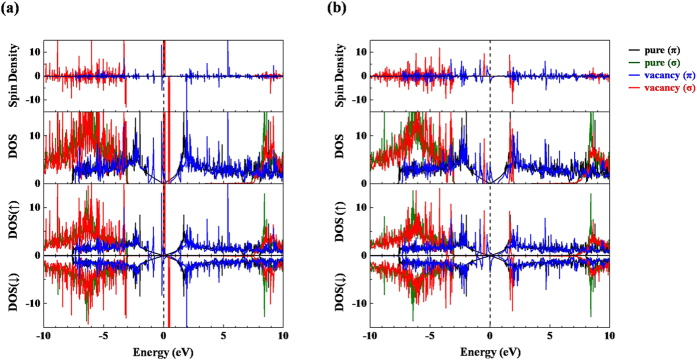
Total DOS, spin-polarized PDOS, and spin density of (**a**) symmetric and (**b**) J-T defect structures. Contributions from π and σ electrons in defect structures are represented in blue and red curves, respectively. Data for pristine graphene are also included for reference. Fermi level (*E*_F_) is set to 0 eV for alignment.

Figure 6(a,b) plot the total DOS, PDOS and spin density that correspond to the defect structures in Fig. 5(a,b) and to pristine graphene as a reference. First, the calculations herein demonstrate that the total DOS close to the *E*_F_ of perfect graphene is dominated by π electron (black curves), agreeing closely with earlier studies^1^,^75^. Similar electronic structures, but with some new features of π character [blue curves in Fig. 6(a)] at 1–3 eV below *E*_F_, are identified in the graphene with a symmetrically distorted vacancy. Also, a rather sharp feature associated with the occupied π orbital (blue curves) next to *E*_F_ reveals the origin of the local magnetic moment and demonstrates that the magnetic property associated with the symmetric vacancy geometry is dominated by π electrons, even though an unoccupied σ orbital (red curves) emerges very close to *E*_F_. However, in asymmetric J-T defect configuration, the J-T splitting redshifts a spin-up σ orbital (red curves) down to 0.5 eV below *E*_F_ and blue-shifts its spin counterpart [the first red peak above *E*_F_ in the spin-down channel of Fig. 6(b)] to 1.7 eV above *E*_F_. This huge J-T splitting of more than 2 eV is responsible for the local magnetic moment at the unsaturated C atom [Fig. 5(b)] adjacent to the vacancy. Therefore, the calculated PDOS demonstrates that the J-T distortion-induced imbalance of carbon σ orbitals close to the defect is critical to enhance local magnetic moments in GO.

In summary, the results of C *K*-edge STXM-XANES provide clear evidence that the higher number of C 2*p*(σ*)-derived defect/vacancies states, rather than of the C 2*p*(π*) states that are bound with oxygen-containing and/or hydroxyl groups on the GO surface, is related to the change of magnetic behavior from that of ferromagnetic GO to that of paramagnetic M-rGO and H-rGO observed from both experimentally and theoretically. The reduction of the width of PL line-shapes and the VB-PES features of C 2*p* states from GO to M-rGO and then to H-rGO further supports the finding that the number of defects decreased as PT-reduction proceeded, importantly, affecting the magnetic behavior of GO sheets. These various magnetic behaviors in GO, M-rGO and H-rGO were verified by the M-H hysteresis curve and XMCD measurements. The spin-polarized DFT calculations of graphene with monovacancy further support the finding that the magnetism originates in defects/vacancies, and in particular that the J-T distortion of the local defect structure is responsible for magnetic moments in GO, as experimentally observed.

## Methods

### Preparation of GO and rGOs

GO was synthesized using the modified Hummers method^76^ and M-rGO and H-rGO were obtained by PT-reduction process of GO. Aqueous GO solutions were placed on a hot (150 °C) Si-substrate and irradiated under a steady-state Xe lamp (500 W) for 3 and 6 hrs. under ambient conditions to prepare M-rGO and H-rGO^27^, respectively.

### Characterizations

Field-emission SEM and TEM were performed to study the effect of photo-thermal treatment on the morphology of GO. Room-temperature M-H hysteresis loop measurements were made using superconducting quantum interference devices magnetometer when a magnetic field was applied in the out-of-plane direction. The VB-PES spectra were collected at the Undulator-09A beamline, at the National Synchrotron Radiation Research Center in Hsinchu, Taiwan. The C and O *K*-edge XMCD were obtained from Beamline-4B of the UVSOR-III Synchrotron of the Institute for Molecular Science, Okazaki, Japan. The C *K*-edge STXM and corresponding XANES spectra were obtained at the SM-beamline at the Canadian Light Source (CLS), Canada. In STXM measurement, a monochromatic X-ray beam was focused using a Fresnel ZP to a ~30 nm spot onto the sample, and the sample was raster-scanned with the synchronized detection of transmitted X-ray to generate a sequence of images (i.e. image stacks) over the range of photon energies of interest. Energy scans of the regions of interest were performed stepwise through with a typical resolving power (*E/*Δ*E*) of ~5000 at the C *K*-edge. Powder samples of GO, M-rGO and H-rGO were solvent deposited on Si_3_N_4_ windows for STXM measurement. The STXM data were analyzed using an aXis2000 (http://unicorn.mcmaster.ca/aXis2000.html) and PCA_GUI (http://xray1.physics.sunysb.edu/data/software.php).

### Theoretical calculations

Spin-polarized DFT calculations of graphene with a vacancy in its supercell were performed using the Vienna *ab initio* simulation package (VASP). The exchange-correlation functional in a generalized gradient approximation (GGA) with the Perdew–Burke–Ernzerhof (PBE) functional was used to approximate electron-electron interactions^77^,^78^,^79^. The kinetic cutoff energy for the plane-wave basis set was set to 400 eV. The monovacancy with a defect concentration of 2% was modeled by removing one C atom from a periodic 5 × 5 supercell of primitive unit cell of pristine graphene. A vacuum space of 15 Å was used to prevent an artificial interaction between the graphene and its periodic imagines. The Brillouin zone integral was sampled on 4 × 4 × 1 Gamma-centered Monkhorst-Pack grid of all supercell calculations^80^. Defect-induced structural distortion was determined by relaxing atomic structures under Hellmann-Feynman forces with a tolerance of 0.005 eV/Å^81^.

## Additional Information

**How to cite this article**: Wang, Y. F. *et al.* Visualizing chemical states and defects induced magnetism of graphene oxide by spatially-resolved-X-ray microscopy and spectroscopy. *Sci. Rep.* **5**, 15439; doi: 10.1038/srep15439 (2015).
